# Supplementary material for: Collaboration in health promotion for newly arrived migrants in Sweden
Source: PLoS One. 2020 May 29;15(5):e0233659. doi: 10.1371/journal.pone.0233659 (PMC7259499; doi:10.1371/journal.pone.0233659)
Supplement: S1 File — (PDF) [file pone.0233659.s001.pdf]

S1 Interview guide

In original language (Swedish)

Frågeguide för fokusgruppsintervjuer med handläggare/specialister anställda vid Arbetsförmedling och kommuner, maj 2016.

Hur har det påverkat dig i din profession att du/din enhet deltagit i de två tidigare studierna?

Ni har nu här strax före intervjun fått ta del av några av resultaten från de två tidigare studierna. Hur upplever ni att det speglar er vardag med möten med människor som har migrerat?

Vilka av de resultat som tidigare har presenterats bedömer ni att är viktigast för era klienters förutsättningar för hälsa?

Upplever ni det som att de resultat som ni bedömer som viktigast är överraskande/nya eller är de mera förväntade och att de nu blivit bekräftande?

Kan ni beskriva vilka allmänna uppfattningar ni har om vad hälsa och hälsofrämjande och vad det innebär för er?

Hur ser ni på skillnaderna i svaren mellan vad som angivits i hälsokartläggningen och i Audit-registreringarna framför allt sociala situationen och levnadsvanor?

Vad anser ni att ligger bakom följande samband och skillnader?

- Samband familjen som resurs och tillfredställande ekonomi
- Skillnader i reflektionerna beroende på om klienten var en kvinna eller en man
- Skillnader i reflektionerna beroende på vilken typ av kommun handläggaren/specialisten tjänstgjorde i

Hur skulle ni vilja att ett forum för externsamverkan och hanteringen av tillgängliga underlag fungerade? I förhållande till uppdraget och sekretess I förhållande till tillgångar och hinder ni upplever att det finns i er vardag

Vad säger era erfarenheter er om att det var sällan förekommande att idéburen verksamhet inkluderades i reflektionerna under temat pågående kontakter? I temat sociala nätverk-fritid/kultur/trossamfund I temat risk – levnadsvanor.

Är det något av det som kommit upp tidigare i diskussionen som du reflekterat särskilt över, positivt eller negativt?

Anser ni att vi har lyft alla de perspektiv kring hur handläggare/specialister definierar hälsofrämjande faktorer med utgångspunkt från de resultat som framkommit i de två föregående studierna?

Är det något ni vill tillägga?

English translation

Interview guide for focus group interviews with authority officials/specialists at the Public Employment Services and municipalities, May 2016.

How has taking part in these studies affected you in your profession?

Before this interview, you were presented some of the results from the earlier part-studies. How did you feel it reflected your everyday work meeting people who have migrated?

Which of the earlier presented results do you think is the most important for your clients' possibilities for good health?

Do you experience the results that are pointed out as most important as surprising or new? Or are they expected and now confirmed?

Can you describe your general view on health and health promotion and what it means to you?

How do you view the differences between what has been stated in the APO-Audit registrations and these registered reflections, especially considering the social situation and lifestyle?

What do you consider being the cause of the following associations and differences:

- Association between family as a resource and satisfying economic situation
- Differences in reflections depending on if the client was a woman or a man
- Differences in reflections depending on the type of authority official

How would you like a forum for external collaboration and handling of client related documentation to work? Regarding your assignment and professional secrecy? Regarding resources and obstacles in your everyday work?

What do your experiences say about the limited presence of civil society in your reflections about ongoing contacts? About the theme social networks, spare time/culture/religion and lifestyle factors?

Is there anything specific from the previous discussion that you have reflected upon more, positively or negatively?

Do you think we have discussed all the perspectives on how authority officials define health promoting factors with the starting point in the results from the earlier part-studies?

Are there anything else you would like to add?
