# Supplementary material for: Collaboration in health promotion for newly arrived migrants in Sweden
Source: PLoS One. 2020 May 29;15(5):e0233659. doi: 10.1371/journal.pone.0233659 (PMC7259499; doi:10.1371/journal.pone.0233659)
Supplement: Attachment — Submitted filename: Response to Reviewers.docx [file pone.0233659.s002.docx]

*Dear Editor and Reviewers,*

*First off, we would like to thank the Academic Editor and the Reviewers for valuable feedback on the article. We are grateful for all the comments and the thorough review of our article. We have processed them all and incorporated almost every point raised in the now re-written article. See our response to the comments in cursive below.*

*We can see how the article benefitted from all the comments and we are happy to see the finished result. Please let us know if there is something we have misunderstood or could further change in the text.*

*Thank you, looking forward to hear from you again.*

*Kind regards,*

*Sara Svanholm, Heidi Carlerby & Eija Viitasara.*

Comments from Academic Editor

Please ensure that your manuscript meets PLOS ONE's style requirements, including those for file naming.

*We have now gone through the style requirements, updated the files accordingly, and gone through the formatting in the article.*

Please address the following:

- Please include additional information regarding the interview guide used in the study and ensure that you have provided sufficient details that others could replicate the analyses. For instance, if you developed a guide as part of this study and it is not under a copyright more restrictive than CC-BY, please include a copy, in both the original language and English, as Supporting Information. In addition, please provide further details concerning the pilot testing of this guide, for example, how many participants were included and from where were they recruited.

*The interview guide (in Swedish as well as in English) has now been added as supporting material. As suggested, we also specified the pilot testing with two officials and one research fellow as well as described the process under the sub-heading data collection.*

- Please provide additional details regarding participant consent. In the ethics statement in the Methods and online submission information, please ensure that you have specified what type of consent you obtained (for instance, written or verbal, and if verbal, how it was documented and witnessed). If your study included minors, state whether you obtained consent from parents or guardians. Thank you for your attention to these queries.

*Verbal consent was collected before each step in the project, so also before the focus group interviews. The head researcher (H.C.) as well as the participating partner official from the County Administrative Board were present and noted the consent. This has now been described under the sub-heading ethical considerations.*

We note that you have indicated that data from this study are available upon request. PLOS only allows data to be available upon request if there are legal or ethical restrictions on sharing data publicly.

In your revised cover letter, please address the following prompts:

a) If there are ethical or legal restrictions on sharing a de-identified data set, please explain them in detail (e.g., data contain potentially identifying or sensitive patient information) and who has imposed them (e.g., an ethics committee). Please also provide contact information for a data access committee, ethics committee, or other institutional body to which data requests may be sent.

b) If there are no restrictions, please upload the minimal anonymized data set necessary to replicate your study findings as either Supporting Information files or to a stable, public repository and provide us with the relevant URLs, DOIs, or accession numbers.

We will update your Data Availability statement on your behalf to reflect the information you provide.

*Yes, we seem to have misunderstood the data availability statement. It should state that all relevant data is included in the article.*

Please amend either the title on the online submission form (via Edit Submission) or the title in the manuscript so that they are identical.

*We have now corrected this. The correct title is Collaboration in health promotion for newly arrived migrants in Sweden.*

Comments from reviewer 1

This study aims to explore the perspectives of local authorities working on a collaboration with health promotion activities in the Establishment Program (EP) for newly arrived migrants in Northern Sweden. Authors suggest an intersectoral approach to health promotion may reduce health inequalities among migrations in Sweden. To achieve their aim, the authors conduct a secondary inductive qualitative content analysis on focus groups with 23 officials (3-7) participants in each group. Findings suggest that EP officials have an unclear conceptualization of their role in providing health promotion activities, and to implement activities, there would need to be structural changes to encourage collaboration. Of particular note, findings point to challenges in developing a coordinated response among multiple agencies and NGOs to meet the full range of needs of newly arrived immigrants to Northern Sweden.

However, this study has the potential to offer insight into the opportunities and barriers to collaboration and implementing health promotion activities into the EP program; currently, the paper lacks conceptual clarity. Issues in the organization of the lit review and methods make it unclear whether this is an evaluation of an existing health promotion intervention, or the authors are exploring the potential of implementing a health promotion activity into EP. If either is the case, there needs to be a stronger argument about the strengths of the EP program as an appropriate point of intervention for health promotion activities. Methods need to be clarified. After a review of the original study, this secondary analysis does not appear to be a duplication – it would benefit from integrating some of the details from the original study in the methods. Of particular note, transparency is needed how this could have affected the research as a limitation and how findings of the data that was offered to the participants prior to the interview.

Major revisions would be needed to accept the manuscript for publication. Further acceptance would need to be conditional that the authors were able to establish the presentation of quantitative results to study participants did not threaten the integrity of the research.

Introduction:

Currently, the order of the key concepts - migration and the social determinants of health, health promotion, and intersectoral approaches – are mixed up. Making it confusing to the reader. Social determinants of health described in this definition reflect only the structural level of the social determinants of health. Since the paper is on migration and health inequality – the authors should consider framing migration as a social determinant of health and how migration contributes to health inequality

*Yes, we see how it could benefit from improvements in the structure. As advised we have now re-written the background to incorporate a perspective of migration and health, but also restructured the entire introduction and added two sub-headings to facilitate reading.*

*Although framing migration as a social determinant of health is one good way of illustrating migrants’ risk of facing inequities in health, we have after careful considerations decided to not use this perspective in the current article. The focus of the current research is on how, within the Establishment Program, actions on the other social determinants of health can promote newly arrived migrants’ health. We, therefore, feel that the current perspective, of how migration risk affect migrants’ social determinants of health, is a better fit for now.*

Limited explanation about how health promotion in the EP program can address these inequalities and why an intersectoral approach is needed.

*To clarify this we now added a paragraph with information and motivation in the introduction, under the sub-heading The Swedish context and the Establishment Program.*

Avoid the use of words like “good” – describe the status of population health among the ethnic majority in Sweden.

*Yes, this is of course right. We re-wrote those sentences.*

Be careful about generalizations about social-economic status and mental health. There is more evidence trauma experienced during the migration process, and persistent exposure to discrimination are thought to contribute to inequalities in mental health or poor rated health.

*In line with comment, we have in our re-structure of the introduction been more careful with the wording.*

The conceptual understanding of the migration health process in this paper is limited. What about the migration health paradox? Newly arrived immigrants often have better health. How could HP activities in the HP program address trajectories of poor health in the country of destination.

*We initially chose to leave out the healthy migrant effect due to its limitations regarding refugees. However, in line with this comment, we have now instead added it, to point out these limitations and added a couple of references on the benefit of early health focus.*

More justification is needed on why the authors selected Northern Sweden as the focus of the study.

*We agree and we have now clarified the reasons for choosing the area Northern Sweden (mainly the specific prerequisite for both migrants and officials) in the background.*

Methods:

In the first paragraph, it is unclear whether HP is an activity that local authorities already engaged in.

*To facilitate a better understanding of the current context we have now explained the context of the Establishment Program more clearly in the background. We also explained how the officials in their everyday work do work with health promoting activities.*

The preliminary quantitative results from the mixed methods study were presented to the participants. This raised methodological concerns about how participants could have been influenced. To amend this problem, the following explanations are needed:

What were the results that were presented?

*We now explained it more clearly under the sub-heading data collection. It was the data on what kind of health promoting activities, and to what extent, they had reflected upon it in meetings with their clients. This to make “health promoting activities” more specific and easier to discuss. This step follows the APO-Audit method, which the main project followed and we have now incorporated this in the method description.*

Given the results that were presented – it is questionable whether this is purely a qualitative study or a mixed-methods study where only the qualitative analysis revised.

*Yes, this is right. The main project used the APO-Audit method and mixed methods, as now described in the method chapter. We removed the “a qualitative study” from the title and restructured the first paragraph in the method to ensure the context is clear.*

Authors cite Graneheim and Lundman – the article specifies decisions in coding regarding latent or manifest content. This needs to be defined.

*It was a latent analysis, which has now been specified.*

A citation is needed for the inductive approach as deductive/inductive content analysis is not specified in the Graneheim and Lundman.

*This has now been corrected. We added the source reference: Patton (2015).*

Findings:

Line 167- this quote does not seem to match the findings.

*We see the problem and have now restructured the paragraph and moved the quote to earlier in the text to ensure that the explanation matched the quote.*

Line 162 – is there evidence guessed other’s role incorrectly?

*It was more about the work other officials did or did not do, and how other organizations and authorities worked. We now added a quote to illustrate this.*

Line 188 – this is an important quote – it would benefit from additional interpretation.

*Yes, we agree, we now added a further explanation of this quote.*

There needs to be clarification on whether there is a clearly defined role for HP activities among these officials. Is the lack of clarity because they do not have an assigned health promotion role? Then this is not a finding. Or is it that they do not know what other actors in the system do?

*We agree that there was some uncertainty regarding this in the article. We have now specified this in the introduction, method, and discussion to clarify how the activities were part of the officials’ everyday work, which is regulated in policies.*

Conclusion:

This section seemed to be the strongest in terms of contributions to the literature.

Authors highlight gaps in knowledge of HP implementation in Northern Sweden but do not return to it fully in the discussion.

*We have now clarified how the area prerequisite and the northern Sweden context impact the results. This was primarily in the municipality structure and service accessibility.*

Line 239 – what did it raise that was in line with the Ottawa Charter?

*We have now clarified the sentence. It was the collaboration aspect in health promotion that was from the Ottawa Charter.*

Who is Corbin, Brännström, Rantala, etc.? A brief summary of the study is needed before referring to how this study contributes to their findings.

*To clarify this, we have now added the context of their articles to highlight the relevance regarding the connection to our research findings.*

Other notes:

Copy editing is needed. There are multiple places where authors split infinitives, and the incorrect tense is used.

*Yes, the article has now gone through a professional language control.*

Avoid beginning a paragraph with the name of the author. There first needs to be a topic sentence.

*We have now restructured the article to accommodate this.*

Comments from reviewer 2

This is an interesting study on collaboration in health promotion for newly arrived migrants.

Remarks:

1. The topic on health among migrants is a large research field, with a great many publications, also in Sweden. The area is very sparsely described, with only a few references, mostly on mental health. Besides, the authors don’t mention the concept “Healthy Migrant Effect”. Thus, a better brief review of possible health problems among migrants is desirable. There are also reports on the health among migrants in Sweden, one older (in Swedish) but still of interest is from Statens Folkhälsoinstitut, 2002:29 (“Födelselandets betydelse”).

*In line with the reviewers’ advice, we have now extended the introduction with a section focused on migration and health. We have introduced the concept of the healthy migrant effect and explained how it has been questioned regarding refugees and stressed the need for early health focused interventions.*

*Instead of adding the “Födelselandets betydelse”, we added a newer report (Health, socioeconomic, and lifestyle factors among foreign-born individuals in Sweden) from the same agency (now Public Health Agency of Sweden) on migrants’ health.*

2. The topic of intercultural consultations in primary care in Sweden has also been studied, e.g. by Rothlind E. et al in 2018 (actually published in PLoS One). Thus, problems correlated to these intercultural consultations should at least be mentioned.

*Very interesting and important article. We have read it, discussed among ourselves, and decided that we cannot include this perspective to the article as of now. In another context, more focused on officials’ relations to their clients, this would have been a both fitting and valuable comparison to make. However, the data in the current material is limited to the relationship between officials. We will take it with us for future ideas though.*

3. The authors mention some methodological considerations. The head researcher conducted the focus group interviews; did anyone else in the team participate? Do the participants represent different aspects beside the different types of municipalities? Did the study reach saturation of results, or could some aspects have been missed?

*Many important questions that we now have specified in the method chapter. It was the head researcher that conducted the interviews and an administrative partner official from the county administrative board also attended. That the municipalities were chosen to represent the different types, but the officials’ backgrounds also differed. Finally, that the interviews reached saturation.*
